# Domain general learning: Infants use social and non-social cues when learning object statistics

**DOI:** 10.3389/fpsyg.2015.00551

**Published:** 2015-05-05

**Authors:** Ryan A. Barry, Katharine Graf Estes, Susan M. Rivera

**Affiliations:** ^1^Department of Human Ecology, University of California, DavisDavis, CA, USA; ^2^Center for Mind and Brain, University of California, DavisDavis, CA, USA; ^3^Department of Psychology, University of California, DavisDavis, CA, USA; ^4^MIND Institute, Davis Medical Center, University of California, SacramentoSacramento, CA, USA

**Keywords:** statistical learning, gaze following, non-social cues, infancy, eye tracking

## Abstract

Previous research has shown that infants can learn from social cues. But is a social cue more effective at directing learning than a non-social cue? This study investigated whether 9-month-old infants (*N* = 55) could learn a visual statistical regularity in the presence of a distracting visual sequence when attention was directed by either a social cue (a person) or a non-social cue (a rectangle). The results show that both social and non-social cues can guide infants’ attention to a visual shape sequence (and away from a distracting sequence). The social cue more effectively directed attention than the non-social cue during the familiarization phase, but the social cue did not result in significantly stronger learning than the non-social cue. The findings suggest that domain general attention mechanisms allow for the comparable learning seen in both conditions.

## Introduction

The natural environment presents infants with multiple streams of information concurrently. They encounter many objects, some moving, some stationary; they hear sounds, some linguistic, some non-linguistic; they see people, some interacting with them, others in the background. Adults regularly direct infants’ attention toward the most important information in the environment by using social cues, including eye gaze and speech. A cue does not need to be social to capture an infant’s attention, however. Non-social cues, such as directional movement, can cause infants to shift attention (e.g., Wronski and Daum, 2014). The current study investigated how social and non-social cues affect learning of a visual statistical regularity.

**FIGURE 1:**
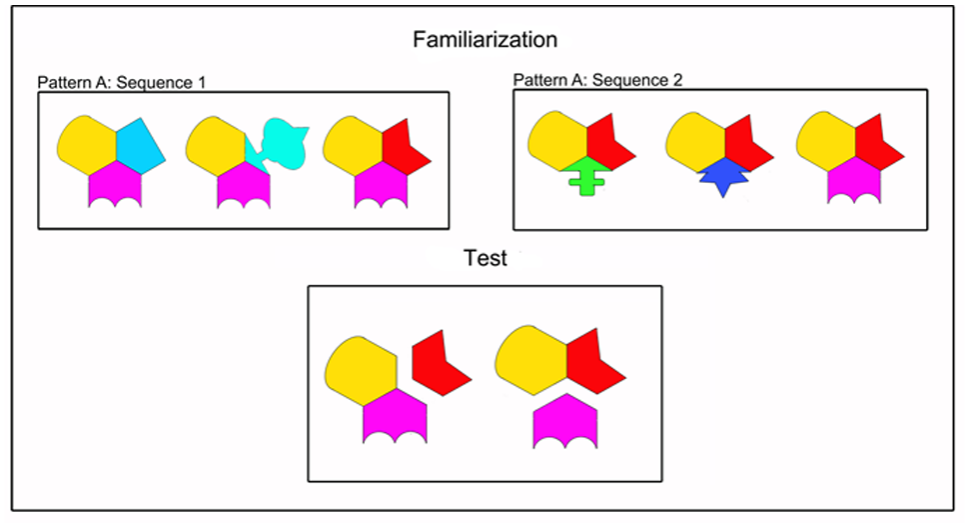
**Example Sequence**. There were three patterns: A, B, and C. Pattern A is shown here. For a more detailed figure, see Wu et al. (2011). Shapes were shown sequentially during familiarization trials. Shapes were shown simultaneously during test trials. The split on the left is consistent with Sequence 1 but inconsistent with Sequence 2. The split on the right is inconsistent with Sequence 1 but consistent with Sequence 2. All stimuli were in full color against a black background.

An infant’s surroundings contain statistical regularities that learners can use to detect structure in a busy environment. For example, infants can detect regularities and co-occurrences in visual shape sequences and scenes (Fiser and Aslin, 2002; Kirkham et al., 2002, 2007). Detecting visual statistical regularities has even been documented in newborns (Bulf et al., 2011). Further, infants have been shown to detect co-occurrences and use them to predict object behavior. In one study, Wu et al. (2011; Experiment 1) exposed 9-month-old infants to sequences of three-shape clusters in which two pieces always co-occurred and one piece constantly changed (see **Figure 1**). They found that infants could keep track of which pieces co-occurred. The infants showed a preference for scenarios in which the co-occurring pieces split apart from each other rather than scenarios in which the co-occurring pieces remained together. Detecting co-occurrences is important for learning about object individuation. For example, a teacup may be seen in multiple situations: with a spoon inside of it, containing a straw, sitting on a saucer, etc. An infant’s ability to recognize that the body of the cup and the handle are always present together, but that a spoon, straw, or saucer only appear along with these pieces occasionally will allow for them to make predictions about the behavior of the object. If the cup is knocked over, the handle and body will likely remain intact but the spoon may move away.

Detecting statistical regularities in the environment is one way in which infants can focus their attention toward potentially relevant information among streams of irrelevant information. A conundrum can arise, however, when more than one stream of information appears to be relevant based on statistical regularities. Infants must then rely on additional cues to guide their attention.

### Social Cues: Learning from Gaze Following

There is a vast literature regarding the influence of ostensive cues (e.g., gaze cues, pointing, gesturing) on infants’ attention and learning (see Carpenter et al., 1998 for an overview of how infants use social cues). By the end of their first year, infants often attend to hands (Yu and Smith, 2013), and are able to reliably follow another’s pointing gesture (Morissette et al., 1995; Deák et al., 2000). During their second year, infants are able to use both gaze and gesture cues to attach labels to objects (Hollich et al., 2000). Eye gaze is a particularly interesting social cue because infants attend to faces, in general, and eyes, specifically, in their first days (Johnson et al., 1991; Farroni et al., 2002). Although there are a variety of social cues available to infants while learning, the current study focuses on gaze following—an early emerging ability.

Even in their first days, infants are sensitive to social information in the environment. Their use of social cues becomes more sophisticated over the first 2 years of life such that social cues can be used to direct learning. Infants can detect eyes from birth and prefer to look at pictures of direct gaze over averted gaze (Farroni et al., 2002). Newborns can even use directional eye movement of a schematic face to locate peripheral targets (Farroni et al., 2004). This ability to follow directional motion may be the basic building block that allows for infants to learn how to learn from complex and highly salient cues like people. By reflexively orienting attention in the direction of motion, infants have the potential to locate the object of another’s attention (see Triesch et al., 2006; Jasso et al., 2012 for more on how basic attentional mechanisms may allow infants to develop the ability to follow gaze). Faces themselves, however, are highly salient. At 3 months of age, infants are more likely to locate a peripheral target cued by eye gaze if the face disappears from the screen after cueing (Hood et al., 1998), indicating that faces are so compelling as to distract infants from the intended target. By 8 months of age, infants are quite skilled at using gaze cues while the face remains on the screen (Wu and Kirkham, 2010). An important difference between a 3-month-old and 8-month-old infant is the amount of exposure they have had to adults directing their attention. It is, therefore, possible that during the first year of life, infants effectively discover how to learn from faces because of their tendency to respond to directional motion coupled with their experience with faces (see Moore and Corkum, 1994; Triesch et al., 2006; Jasso et al., 2012 for more on how positive reinforecement allows for more sophisticated gaze following to devleop).

By 4 months, neural (event related potential) and behavioral evidence suggests infants use the gaze direction of a social partner to guide attentional resources toward relevant objects (Reid et al., 2004; Reid and Striano, 2005). During the second year of life, infants respond to the joint attention bid of an adult when determining the referent of a label in the presence of two possible referents (Moore et al., 1999; Houston-Price et al., 2006). These studies suggest that infants use adults’ joint attention bids to guide their own attention and learning of surface characteristics and labels.

Infants have more to learn, however, than labels and surface characteristics. They must also attend to and learn many complex visual events in their first year of life, such as visual feature co-occurrences for object individuation and binding sights and sounds to form a complete representation of events. Eight-month-old infants can use a directional head turn to associate a sound with a specific spatial location (Wu and Kirkham, 2010; Experiment 1). Nine-month-old infants take advantage of social cues to direct their learning toward relevant patterns in the presence of irrelevant distracting patterns (Wu et al., 2011; Experiment 4). These studies all indicate that social cues have the ability to direct attention and help infants select the appropriate information to learn.

### Learning from Non-Human or Non-Social Cues

Although much research has investigated how following a social cue allows infants to gather and use information early in life, we know little about how social and non-social cues differentially guide learning. By the end of their first year, infants have had a lot of experience with faces guiding attention and learning. Infants are unlikely to have had such extensive experience with non-social cues guiding learning; objects in the environment have likely attracted much attention but not often directed attention explicitly. The novelty of relying on a non-social directing cue could make learning in this context more difficult.

Studies examining the use of non-social cues have produced mixed evidence regarding their effectiveness. Two different studies found that non-human objects can direct infants’ attention when they possess the biologically relevant characteristics of having a face or responding contingently to the infant’s own behavior but not when both of these characteristics are absent (Johnson et al., 1998; Deligianni et al., 2011). Although possessing a face and responding contingently to infants’ behavior appear to be sufficient for directing attention, these characteristics do not appear to be sufficient for promoting word learning (O’Connell et al., 2009). O’Connell et al. (2009) compared the effectiveness of a robot and a human on gaze following and label learning in 18-month-old infants. Both the robot and human teacher effectively directed infants’ attention to the target objects, but infants only succeeded in learning the label when taught by a human.

Exogenous, peripheral cues can also direct attention. Salient peripheral cues, such as flashing lights, can attract the attention of infants (Colombo, 2001). Wu and Kirkham (2010) compared the effectiveness of a central, social cue (an adult turning toward one location), and a peripheral, non-social cue (flashing squares around one location) for directing infants’ attention to one of two identical audiovisual events. While both cues effectively directed more attention to the cued event than the un-cued event, infants only displayed learning of the event marked by the social cue. They did not seem to learn the event marked by the non-social cue. Therefore, the authors concluded that central, social cues produce a greater depth of learning than peripheral, non-social cues. The authors acknowledge, however, that the cues used in their study differed fundamentally in multiple ways (central and endogenous vs. peripheral and exogenous). Thus, it could not be concluded what aspect of the social cue enhanced learning.

Wahl et al. (2012) used only central cues and compared how social (adult head turn) and non-social (a car turn) cues differentially affect encoding of surface characteristics of objects. The cues were comparable in motion and placement of salient characteristics (two eyes above a mouth and two headlights above a fender). Infants watched the central cue turn toward one of two novel objects. At test, four-month-old infants showed a novelty preference in the social condition. The infants exhibited no significant looking preference with the non-social car cue, indicating that they encoded the cued object more deeply with the presence of the face than with the presence of the car.

The research reviewed has indicated that non-human cues that possess human-like characteristics are able to direct attention, but it is still unclear if attention can be directed by non-social cues without human-like characteristics during the first year. The central, non-social, or non-human cues used in previous studies (i.e., cars and robots) may have been too perceptually interesting to allow for sufficient “gaze” following and learning. Because a highly salient face distracts attention in infants with little experience with such cues (Hood et al., 1998), it is possible that a similar phenomenon occurs with unfamiliar, salient non-human cues. That is, an infant may still have a tendency to shift attention in response to the directional motion of a non-social cue, but the saliency of the non-social cues used in previous studies may have prevented infants from using the theorized basic building block of responding to directional motion to effectively utilize the cue to direct attention and learning. Therefore, the previous studies may not provide an ideal comparison as to how infants learn from social and non-social cues.

A less interesting, non-social directing cue may be able to effectively direct attention and allow for deep processing. A simple cue, such as an arrow, may allow for this. Varga Jakobsen et al. (2013) investigated the use of arrow cues during infancy and found that while infants are limited in their ability to orient their attention using arrows, perceptual weight more strongly cues attention than the direction of an arrow. That is, infants orient their attention in the direction of a perceptually heavy, non-directional “arrowhead” (a square) better than they orient to a directional arrowhead. Varga Jakobsen et al. (2013) used static cues, however, and motion likely attracts more attention than static cues during infancy (Colombo, 2001). Therefore, a dynamic arrow moving toward an object should more consistently direct attention in the appropriate direction. A dynamic arrow could potentially eliminate the distraction introduced by complex, non-social cues. By removing the complex characteristics (e.g., the headlights and fender of a car) of the cue but retaining the theorized basic building block (i.e., directional motion), we can begin to determine if this is in fact the basic building block that allows for infants to follow another’s gaze.

### The Current Study

The current study investigates how 9-month-old infants use social and non-social cues to orient their attention and learning. We chose to test 9-month-olds because infants this age reliably respond to and use gaze cues (e.g.,Senju et al., 2006; Striano et al., 2006; Gredebäck et al., 2008).

This study has two motivating questions. First, can a perceptually uninteresting, non-social, central cue successfully direct attention during the first year? Second, can this type of non-social cue promote learning as effectively as a social cue? It has been theorized that social cues are privileged and therefore more effective for learning (Wu and Kirkham, 2010; Wahl et al., 2012). But a cue may just need to possess certain properties such as familiarity, simplicity, or motion, to enable learning.

**FIGURE 2:**
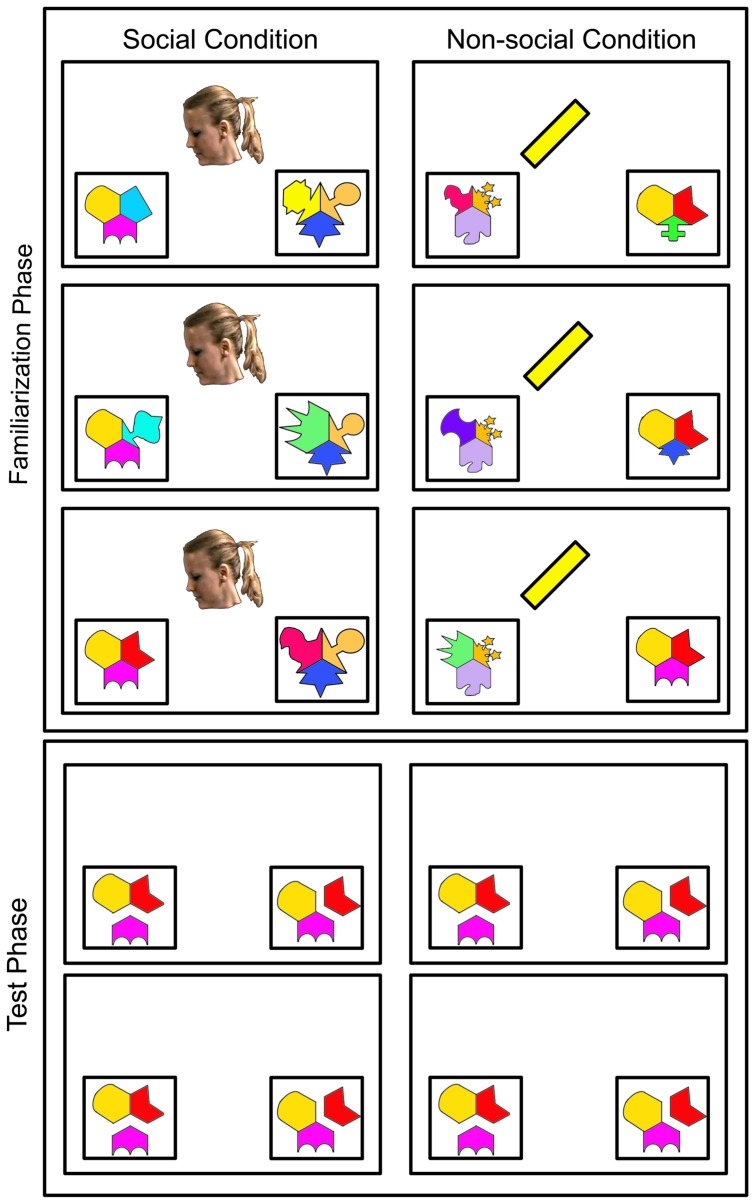
**Example Stimuli**. Examples of stimuli for infants in the social and non-social conditions. Stimuli were in full color against a black background.

We used a perceptually uninteresting (i.e., consisting of no visual pattern or human-like features) solid yellow rectangle (see **Figure 2**) as our non-social cue to guide learning. The rectangle rotated 45∘ so that it was perceptually weighted toward one side of the screen. This makes the cue much like an arrow, such that an infant would follow the orientation of the shape down to the target object. Because of an arrowhead’s symbolic nature, and the lack of evidence suggesting that infants orient to arbitrary symbols (e.g., Varga Jakobsen et al., 2013), we used only the arrow shaft. We hypothesized that the cue would effectively orient attention without prior experience because it would reflexively pull attention to the side of the screen toward which the rectangle rotated. The cue also possessed no human-like characteristics because we speculated that such interesting cues might attract attention that should be devoted to the to-be-learned information.

We investigated how infants use social and non-social cues to guide their learning of relevant streams of information in the presence of irrelevant streams. Specifically, we tested whether infants can form predictions about how objects behave based on feature co-occurrences. We selected a visual statistical learning task because it provided the needed context of two competing, equally informative and attention-capturing streams of information. Within this context, the social or non-social cue emerges as the only information the infant can use in order to guide her attention and learning toward one stream of information over the other. Previous research has demonstrated that infants use social (head turn with eye gaze) cues to guide attention and learning in visual statistical learning (Wu et al., 2011); it is not yet clear whether non-social cues also support attention and learning.

As in Wu et al.’s (2011) experiment (Experiment 4), all infants saw two different sequences of shapes simultaneously in the bottom right and left corners of the screen. Half of the infants were directed to the target sequence by a social cue (a person turning toward one sequence) and half of the infants were directed by a non-social cue (a yellow rectangle rotating toward the target sequence). Each sequence consisted of a set of 3 three-piece shapes with a statistical regularity: two pieces of each shape had a high probability of co-occurring (probability = 1.0) and the other piece had low probability of co-occurring with either of the other two pieces (probability = 0.33). Infants viewed a continuous sequence of the shapes, presented in a randomized order, to provide them with the opportunity to detect the regularities. At test, infants saw events in which pieces split apart from each other. During a consistent split, the pieces with high co-occurrence probability split apart from the piece with low probability. During an inconsistent split, the pieces with high probability split apart from each other, leaving one piece paired with the low-probability piece. If infants learned the properties of the target sequence during the familiarization phase, then they should display a looking time preference for one of the splitting events at test. Wu et al. (2011) found that infants attended longer to the inconsistent splitting events, and we expected to replicate this pattern in the social cue condition that was based on their design. However, any reliable discrimination of the consistent and inconsistent splits indicates that infants learned the sequences that were presented during familiarization. We used eye-tracking technology to measure visual attention and gaze patterns during familiarization and test events.

We predicted that the social cue would not direct attention toward the target sequence more effectively than the non-social cue because infants should reflexively orient to directional motion in both conditions. We also predicted that the social cue would not result in significantly stronger learning than the non-social cue because the simplicity of the non-social cue would not distract attention away from the to-be-learned information.

If the non-social cue works as well as the social cue, then this may suggest that a domain general response to directional motion drives performance in both conditions. If the non-social cue does not work as well as the social cue, however, then simple directional motion may not be the starting point for gaze following.

## Materials and Methods

### Participants

A total of 55 healthy, full-term infants (28 females) comprised the final sample: twenty-eight infants (15 female) in the social condition (mean age = 9 months 21 days; age range = 9 months 5 days to 10 months 5 days) and twenty-seven infants (13 female) in the non-social condition (mean age = 9 months 19 days; age range = 9 months 5 days to 10 months 9 days). Thirty of the infants were Caucasian, 2 were Asian, 1 was American Indian, 15 were mixed race, and race was not reported for 7 infants. Across these racial groups, 14 were reported to be Hispanic. All of the mothers had graduated from high school, except one whose highest level of education was completion of eighth grade. Seventy three percent of the mothers had earned at least a bachelor’s degree. An additional four infants were tested but excluded from the final sample due to a failure to complete at least three blocks of trials. We excluded two infants as outliers due to their mean preference at test being greater than 2 SDs from the group mean. Infants were recruited through letters to families in the surrounding area and given a toy for participating. The Institutional Review Board approved the experimental protocol, and informed consent was obtained from a parent or caregiver of each infant.

### Apparatus and Stimuli

Stimuli were presented on a 17-inch Tobii 1750 LCD binocular eye tracker (1280 × 1024 pixels resolution) to record infants’ fixations during the task. Eye tracking data were collected at a sample rate of 50 Hz. The average accuracy of the recorded eye coordinates was about 0.5∘, which is approximately 0.5 cm at a viewing distance of 60 cm. The average accuracy in timing was 25–35 ms. Drifts are compensated with an average error of 0.5∘. When one eye could not be measured, data from the other eye were used to determine the gaze coordinates. Data were filtered using Tobii fixation filter with a fixation radius of 35 pixels (0.9∘). Missing data due to blinks (defined as a data loss of 75 ms or less) was interpolated using the gap fill in algorithm within the Tobii fixation filter. The recovery time to full tracking ability after an offset was about 100 ms. Stimuli were displayed using Tobii’s Studio software (Tobii Technology, Sweden), and sounds were presented through external speakers.

The shape animations were created with Macromedia Director MX 2004 and shared by Wu and Kirkham (2010). The video clips were assembled using Final Cut Pro (Apple Inc., Cupertino, CA, USA).

#### Shape Stimuli

Two sequences of shapes (Sequence 1 and Sequence 2) cycled through their patterns in two white boxes in the bottom corners of a black screen. Each sequence consisted of three patterns of shapes (Patterns A, B, and C), each pattern consisted of three shapes, and each shape consisted of three brightly colored pieces. Within each shape there was a statistical regularity such that two pieces always co-occurred (100% chance of co-occurrence) and the third piece changed with the appearance of a new shape in the pattern (33% chance of co-occurrence with either of the other two shapes). Thus, infants could generate expectations about the behavior of the pieces of the shapes. This same regularity was present in Sequence 1 and Sequence 2, but the combination of pieces differed (see **Figure 1**). Each shape appeared at a minimum of 2.1 × 2.5 cm (subtending 2.01∘ × 2.39∘ visual angle) and grew to a maximum of 5 cm × 6.6 cm (subtending 4.77∘ × 6.30∘ visual angle) before the next shape appeared. The presentation order of shape combinations was randomized across participants, and shapes were always presented in the same orientation.

#### Cue Stimuli

The stimuli and timing for the social condition were nearly identical to those used by Wu et al. (2011). All stimuli were against a black background. For the familiarization trials, infants were presented with a centrally located video of a Caucasian woman with a local American accent who looked straight ahead, said “Hi baby! Look at this!,” and then turned her head to look toward one of two empty white boxes at the bottom corners of the screen. The side was counterbalanced between participants, such that the cue turned either to the left or right corner of the screen. The woman wore her hair in a ponytail and a black drape covered her shoulders. Thus, only her head was visible against the black background (see **Figure 2**). Once the head turn was complete, the pattern sequences began (described in the previous section; see **Figure 1**) in each of the white boxes. The sequence that was gazed upon by the woman will be referred to as the target sequence, and the sequence that was not cued will be referred to as the distractor sequence. For half of the participants, Sequence 1 was the target sequence and Sequence 2 the distractor, and vice versa for the other half. After the sequences ended, the woman turned her head back toward the center and the trial ended. Therefore, the woman was present for 3 s prior to the start of the sequences and 2 s after the end of the sequences. Each familiarization trial lasted 11 s. The 11.8 cm × 9.8 cm social cue subtended 11.23∘ × 9.34∘ visual angle.

The timing and location for the non-social condition were similar to those in the social condition, but the woman was replaced with a solid yellow rectangle (8.5 × 1.5 cm, subtending 8.10∘ × 1.43∘ visual angle) that remained upright and stationary for 3 s while music played (in place of “Hi baby! Look at this!”), and then turned 45∘ toward the target (see **Figure 2**). The shape sequence animations then began playing. After the sequences finished, the rectangle turned 45∘ back to the original position.

#### Test Stimuli

The test trial stimuli were identical to those used by Wu et al. (2011). For the test trials (used in both conditions) there were once again two white boxes located at the bottom corners of the screen. Each box contained a different type of split. On one side, the pieces split apart in a consistent manner (in accordance with the statistical regularity presented in the target sequence), such that the pieces with a 100% co-occurrence remained together and the piece that changed broke from those two at a 45, 180, or 270∘ angle (relative to the vertical). On the other side of the screen, the pieces split apart in an inconsistent manner (in violation of the statistical regularity presented in the target sequence), such that the pieces with a 100% co-occurrence split apart from each other where one piece remained with the piece with which it had a 33% co-occurrence and the other piece broke from those two. Each cluster of shapes appeared at a minimum of 2.5 cm × 2.9 cm (subtending 2.39∘ × 2.77∘ visual angle) and grew to a maximum of 6.5 cm × 7.3 cm (subtending 6.20∘ × 6.96∘ visual angle) before the pieces split from each other. Note here that a split consistent with the target sequence was inconsistent with the distractor sequence, and vice versa (see **Figure 1**). Three splits were presented per trial (one per pattern) with each split lasting 3.5 s. Each test trial, therefore, lasted 10.5 s. The locations of the splits during test were counterbalanced between infants such that an inconsistent (or consistent) split could be on either the right or left side of the screen. Finally, the shapes in the splits were presented in four counterbalanced orders for each infant.

### Procedure

Infants sat in their caregiver’s lap 60 cm from the eye-tracker monitor in a small, quiet, dark room. The caregivers were instructed to refrain from interacting with their infants during the experiment. The experiment began with a five-point calibration routine. There were then four blocks of trials with each block consisting of six familiarization trials (two per shape pattern, where each pattern consisted of three shape combinations) and two test trials (each consisting of three splits—one per shape pattern). The familiarization trials were always presented in the same order within a participant. There were four different combinations of test trials within a participant, and two were presented during each block. Therefore, Blocks 3 and 4 are repeats of Blocks 1 and 2. An attention getter (a stuffed animal holding balloons) played between each familiarization and test trial for up to 6 s.

### Data Preparation

Areas of interest (AOIs) were created using Tobii’s Studio analysis software to calculate total looking time to each object on the screen. For familiarization trials, the AOIs were the cue (social or non-social), target sequence, and distractor sequence. For test trials, the AOIs were the box including the consistent splitting events and the box including the inconsistent splitting events. To measure learning, we calculated proportional difference scores (DSs) by subtracting the duration of looking at inconsistent splits from the duration of looking at consistent splits and then dividing that value by the total time spent looking at either split. If the infant did not fixate any of the AOIs during a trial (i.e., total looking time for the trial equaled 0 s), then that trial was removed from the analysis.

## Results

### Familiarization Phase

For the familiarization phase, average looking time (in seconds) was entered into a repeated-measures ANOVA with one within-subjects factor: AOI (cue, target sequence, or distractor sequence); and one between-subjects factor: condition (social or non-social). The analysis revealed no significant main effect of condition, *F* < 1. Infants in the social condition and non-social condition did not differ in the average amount of time they looked at the screen. There was, however, a significant main effect of AOI, *F*(2,106) = 19.90, *p* < 0.001, $\eta_{p}^{2}$ = 0.27. There was also a significant AOI by condition interaction, *F*(2,106) = 33.58, *p* < 0.001, $\eta_{p}^{2}$ = 0.39.

**FIGURE 3:**
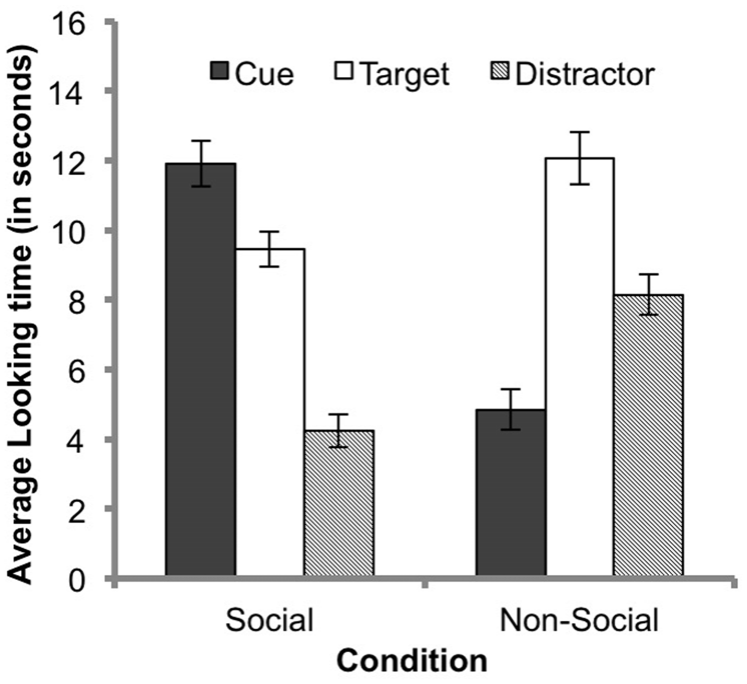
**Average looking time during the familiarization phase for each condition**. Error bars represent SEs.

Because of the significant AOI by condition interaction, simple effects were investigated by performing a separate independent samples *t*-test for each AOI. The average times that infants looked to each AOI is shown in **Figure 3**. Infants spent a significantly greater amount of time looking at the cue in the social condition (*M* = 11.88 s, SE = 0.72) than the non-social condition (*M* = 4.80 s, SE = 0.60), *t*(53) = 7.57, *p* < 0.001, *d* = 2.08. This indicates that the social cue was more interesting to visually explore than the non-social cue. Infants spent significantly less time looking at the target sequence in the social condition (*M* = 9.54 s, SE = 0.54) than the non-social condition (*M* = 12.19 s, SE = 0.81), *t*(53) = -2.75, *p* < 0.01, *d* = 0.76. Infants also spent less time looking at the distractor sequence in the social condition (*M* = 4.43 s, SE = 0.52) than the non-social condition (*M* = 8.08 s, SE = 0.62), *t*(53) = -4.54, *p* < 0.001, *d* = 1.25. This indicates that infants spent a greater amount of time visually exploring both sequences in the non-social condition than the social condition.

Because infants in the social condition spent such a large portion of their total looking time looking at the cue (47%) in comparison to infants in the non-social condition (19%), comparing the mean looking time to target and distractor is not very informative. We can better understand how effectively attention was directed to the target sequence by comparing the proportional DSs (calculated by subtracting the total amount of time spent looking at the distractor sequence from the total amount of time spent looking at the target sequence and then dividing that value by the total time spent looking at either sequence) for each condition. Therefore, we performed an independent samples *t*-test to compare the DS during familiarization in the social and non-social conditions. The DS gives us a measure of how much more the infants are looking to the target than the distractor sequence. The average DS for infants in the social condition (*M* = 0.38, SE = 0.06) was significantly greater than the average DS for infants in the non-social condition (*M* = 0.19, SE = 0.06; *t*(53) = 2.41, *p* < 0.02, *d* = 0.66), indicating that the social cue more effectively directed attention toward the target sequence in comparison to the non-social cue. We also compared the DSs to chance (where the chance DS would be zero) in both conditions in order to determine if infants spent a greater proportion of time visually exploring the target sequence than the distractor sequence during familiarization in both conditions. A significant preference for the target sequence was found in both the social and non-social conditions, *t*(27) = 6.33, *p* < 0.01, *d* = 1.19 and *t*(26) = 3.12, *p* = 0.01, *d* = 0.76, respectively. Therefore, we can conclude that both the social and non-social cues effectively directed attention toward the target and away from the distractor sequence.

### Test Phase

To examine infants’ learning, we calculated a proportional looking time DS, defined as looking time to the consistent split minus looking time to the inconsistent split divided by the total time spent looking at either split. We examined whether infants’ DS differed across block, if the preference differed by condition, and finally if the preference differed based on congruency of familiarization and test locations (see **Table 1** for average DSs across block and condition). Therefore, we entered the DS at test into a repeated-measures ANOVA with one within-subjects factor: block (1, 2, 3, or 4) and two between-subjects factors: condition (social or non-social) and congruency (location of target sequence and test events; congruent, incongruent). The analysis revealed no significant main effect of condition, *F* < 1, no significant main effect of congruency, *F* < 1, no significant main effect of block, *F*(3,111) = 1.11, *p* = 0.35, $\eta_{p}^{2}$ = 0.03, no significant condition by congruency interaction, *F* < 1, no significant block by condition interaction *F*(3,111) = 1.73, *p* = 0.17, $\eta_{p}^{2}$ = 0.05, no significant block by congruency interaction, *F*(3,111) = 1.33, *p* = 0.27, $\eta_{p}^{2}$ = 0.04, and no significant block by condition by congruency interaction, *F*(3,111) = 1.32, *p* = 0.27, $\eta_{p}^{2}$ = 0.04. The absence of a main effect of condition suggests that the strength of learning did not differ in the social and non-social conditions. The absence of a main effect of block suggests that the overall strength of learning did not differ across the experiment, and the absence of a block by condition interaction suggests that this was the case for both conditions.

**Table 1:** Difference score (DS) during test phase.

Condition	Block 1 DS	Block 2 DS	Block 3 DS	Block 4 DS	Average DS
Non-social	-0.02 (0.07)	0.08 (0.08)	0.01 (0.08)	0.25 (0.07)	0.06 (0.06)
Social	0.07 (0.04)	0.12 (0.06)	0.08 (0.08)	0.08 (0.05)	0.09 (0.04)

*Data are reported as mean (SE). The DS was calculated by subtracting the total amount of time spent looking at the inconsistent events from the total amount of time spent looking at the consistent events and dividing that value by the total time spent looking at either event. A negative score indicates a preference for inconsistent events, and a positive score indicates a preference for consistent events*.

If infants show a significant preference for one type of split (consistent or inconsistent) over the other type, we can then conclude that they learned the co-occurrences present in the shape sequences. To determine if there was a significant learning effect, we performed a one-sample *t*-test on the average DSs at test. Because no effect of condition was found in the previous analysis, the average DS across conditions was used (*M* = 0.08, SE = 0.03). When we compared the DS to chance (where the chance DS would be zero, indicating that the infants looked at both sequences for an equal amount of time), we found a significant preference for consistent splitting events, *t*(54) = 2.27, *p* = 0.03, *d* = 0.31. Therefore, we can conclude that infants learned the statistical regularity presented to them.

### Exploratory Analysis

**FIGURE 4:**
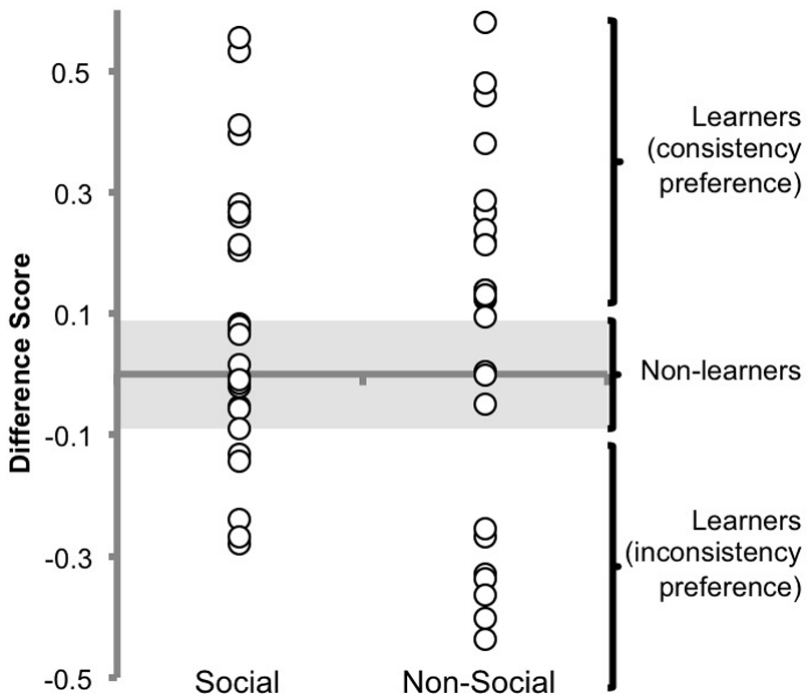
**Average difference score (DS) across the experiment for all infants**.

No effect of condition was found during the test trials, but in order to better characterize the similarities and differences between the social and non-social conditions, the data will be described in more detail here. A scatterplot of the average DSs by condition can be found in **Figure 4**. As previously mentioned, a preference for either consistent or inconsistent splits can be considered evidence of learning in this paradigm because either preference suggests that infants are able to differentiate the two types of splits (see Hunter and Ames, 1988). Because some infants prefer consistent sequences and some prefer inconsistent sequences in both conditions, we categorized infants as learners and non-learners (regardless of their preference) to better represent performance (see **Figure 4**). We calculated the mean of the absolute value of the significant DS found in Wu et al. (2011; -0.09) with the absolute value of the significant DS found in the current experiment (0.08) to determine a threshold for learning. The absolute value of each infant’s average DS was then compared to the threshold. Infants with a score greater than or equal to 0.09 were classified as learners. Infants with a score less than 0.09 were classified as non-learners. Using this criterion for learning, 16 of 28 infants in the social condition can be categorized as learners, and 23 out of 27 infants in the non-social condition can be categorized as learners. We performed a chi square test of homogeneity on these frequencies. The results suggest that the proportion of infants who learned in the social condition differs from the proportion of infants who learned in the non-social condition, χ^2^(1, *N* = 55) = 6.98, *p* < 0.01. More infants learned in the non-social condition than the social condition. Because of the exploratory nature of this analysis, the result should be taken with caution. Regardless, this additional analysis does further support our finding that a social cue does not work better than a non-social cue.

## Discussion

The results of this study suggest that although attention is more strongly directed by a social cue than a non-social cue, there is no significant advantage of using a social cue to guide learning in 9-month-old infants. The lack of a significant difference between the strength of learning in the social and non-social conditions provides support for a domain general learning mechanism. This is the first study, to our knowledge, that shows comparable learning from a social and non-social cue when learning visual statistical regularities in the environment.

The allocation of attention during familiarization differs based on the type of cue used to direct attention. In the present experiment, infants in the social condition spent a greater proportion of time looking at the target sequence in comparison to the distractor sequence than infants in the non-social condition. This indicates that the infants were more strongly directed by a social cue than a non-social cue during familiarization. One could conjecture that since infants are more strongly directed by a person than a moving rectangle, people are inherently better attention-directing cues. However, this could be driven purely by familiarity. Nine-month-old infants have had extensive experience attending to social cues, such as faces, to guide attention outside of the laboratory. The familiarity of using faces to guide attention could account for why infants in the social condition were more strongly directed to the target sequence (in comparison to the distractor sequence) than infants in the non-social condition.

A domain-general attention mechanism driven by familiarity and perceptual saliency could account for why the differing allocation of attention by condition during familiarization resulted in similar learning in both conditions. There appears to be no learning advantage to being more strongly directed to the target sequence or exploring the target sequence for a longer amount of time, so perhaps some threshold exists that infants have to reach in order for learning to occur. If true, then it should make no difference that the presence of a face draws attention to itself as long as the face still directs attention to the to-be-learned information for *enough* time. The infants look to the screen for a comparable amount of time in both conditions, so the most salient object on the screen could simply drive the allocation of attention. For the social condition, the face (not surprisingly) attracts the most visual attention. Faces are highly salient to infants; they prefer to look at faces over other objects (Johnson et al., 1991). For the non-social condition, the target sequence attracts the most visual attention. Infants reflexively orient to this sequence, and because it is more interesting than the orienting rectangle itself, infants spend the majority of their time visually exploring it. Therefore, simple domain-general perceptual saliency and familiarity, rather than the meaning of the stimuli, likely drives attention in the same manner in both conditions during familiarization.

Continuing with the idea of a domain-general attention mechanism, we propose that the current non-social cue was successful at orienting attention because, while novel, it possessed no complex or human-like characteristics. This allows infants to utilize the cue’s directional motion to direct attention. Our non-social cue did not have to be learned before it could be used (i.e., we did not have to teach infants how to use the cue); therefore, the lack of salient characteristics appears to be beneficial for infants. Basic attentional processes could explain why our non-social cue allowed learning to occur, while others in the past have failed. Early in infancy, exogenous attention drives where infants attend, while bottom–up mechanisms result in reflexive orienting to salient stimuli (Colombo, 2001). Therefore, during the first year, any object that is perceptually weighted in one direction should allow for infants to exogenously orient toward the weighted direction. Endogenous (voluntary) orienting is supported by the frontal cortex and emerges later in the first year of life. Therefore, a non-social directing cue that is both perceptually weighted in one direction and perceptually interesting itself (like those used in previous experiments; O’Connell et al., 2009; Wahl et al., 2012), will likely direct attention exogenously initially but an infant nearing her first birthday will be able to endogenously orient her attention to the directing cue itself to explore its salient characteristics. Because of this, a non-social directing cue that is perceptually weighted in one direction but largely uninteresting itself, such as the one used in this study, should effectively orient attention and allow for learning to occur. There are a number of factors that likely contributed to why this non-social cue supported learning. We proposed that it is because it is a bright color (so that it is easily detectable), but not distracting. The motion, color, brightness, and contrast with the background all could have contributed to the effectiveness of the cue. The exact mechanism cannot be determined from this experiment. However, the evidence from the present study suggests that learning was as effective from this simple (but bright) cue as from a complex social cue with many other salient features (i.e., eyes, facial features, hair).

The explanation we have provided for the effectiveness of non-social cues may seem to predict that learning should not occur in the presence of a salient face because faces draw attention to themselves. However, faces have other features that support infants’ use of them as attentional and learning cues. One major feature is familiarity; because infants have extensive experience with faces, they may gather enough experience to learn how to learn from faces early in development. Thus, even when presented with novel faces in novel (lab) settings, they take advantage of social cues. The lack of experience with learning from non-social cues outside of the lab could make this process work slightly differently for familiar social cues and non-social cues. However, with development and additional experience interacting with the world, infants, and children should become more adept at using non-social cues to direct attention and learning (e.g., Kanda et al., 2004; Yoshida, 2012). Future studies should vary the characteristics of non-social cues at different ages to better determine which characteristics facilitate learning and which complicate learning throughout the lifespan.

Why can infants learn equally well from a social and non-social cue? If we consider a basic attention explanation of gaze following, then the results are not surprising. Triesch et al. (2006) proposed that infants’ attraction to faces, coupled with a tendency to shift gaze in response to directional motion (see Farroni et al., 2000), allows for infants to essentially look at the object at which an adult looks. With repeated exposure to faces directing attention, infants’ ability to learn from faces (a highly salient cue) likely improves. When infants are shown an uninteresting, non-social, non-human cue with directional motion, they are able to look at the object at which that non-social cue points. When the directing cue is not particularly interesting to visually explore, learning how to learn from the cue is not a necessity. Therefore, when we remove all of the salient features of a cue (whether social or non-social), it is still as effective as a human face (a cue that infants use regularly).

Finally, the current results revealed a preference for consistent splits, rather than the splitting events that violated their expectations about the shape probabilities. They preferred to look at the events consistent with what they learned, displaying a familiarity preference. The familiarity preference has been suggested to indicate less advanced processing than the preference for events that violate an expectation (Hunter and Ames, 1988; Houston-Price and Nakai, 2004). In habituation paradigms, for example, infants typically show a familiarity preference early in the experiment and then with additional exposure, show a novelty preference. The consistency preference we found differs from the inconsistency preference seen by Wu et al. (2011). However, Fiser and Aslin (2002) found a familiarity preference for co-occurring shapes in their visual statistical learning experiment. Furthermore, it is important to note that a preference for either the consistent or the inconsistent splits indicates successful learning (Roder et al., 2000; Houston-Price and Nakai, 2004). Minor differences in the social cues used across experiments could have led to the consistency preference in the present experiment versus the inconsistency preference found by Wu et al. (2011). For example, the video of the social cue was slightly smaller than the shape sequences in the current experiment, whereas the video was the same size as the shape sequences in the Wu et al. (2011) experiment.

While we are beginning to understand the characteristics of social cues that affect gaze following in nine-month-old infants (e.g., eye contact, the familiarity of the person; Senju and Csibra, 2008; Gredebäck et al., 2010), we know very little about the characteristics of social cues that affect *learning*. Further, although gaze following and learning may be directly related in some circumstances, the results of the current study suggests that stronger gaze following does not directly relate to stronger learning. Future studies should manipulate the features of social cues to better understand how specific features enhance or impede learning.

Additionally, gaze cues are just one of many social cues that infants encounter. Although it is particularly informative to understand the basic perceptual driving force behind gaze following because of its early emergence in infancy, more research is necessary to understand how experience and cognitive abilities interact to allow for a developing child to take advantage of the abundance of social cues (e.g., gestures, head turns, eye gaze, points) in her environment.

## Conclusion

We found that a cue does not have to be social in order to direct infants’ attention and learning. A perceptually uninteresting, non-social cue is just as effective as a human face when guiding learning of a visual statistical regularity. Social cues may be more effective than non-social cues when directing attention during familiarization, but this differing allocation of attention does not appear to affect learning. The saliency of a human face paired with the familiarity of using people to guide learning likely underlies the performance differences seen during familiarization. Domain general attention orienting likely allows for similar performance at test.

## Conflict of Interest Statement

The authors declare that the research was conducted in the absence of any commercial or financial relationships that could be construed as a potential conflict of interest.

## References

[B1] Bulf H. Johnson S. P. Valenza E. (2011). Visual statistical learning in the newborn infant. *Cognition* 121 127–132 10.1016/j.cognition.2011.06.010 21745660

[B2] Carpenter M. Nagell K. Tomasello M. Butterworth G. Moore C. (1998). Social cognition, joint attention, and communicative competence from 9 to 15 months of age. *Monogr. Soc. Res. Child Dev.* 63 1–143 10.2307/1166214 9835078

[B3] Colombo J. (2001). The development of visual attention in infancy. *Annu. Rev. Psychol.* 52 337–367 10.1146/annurev.psych.52.1.337 11148309

[B4] Deák G. O. Flom R. A. Pick A. D. (2000). Effects of gesture and target on 12- and 18-month-olds’ joint visual attention to objects in front of or behind them. *Dev. Psychol.* 36 511–523 10.1037/fflOI 10902702

[B5] Deligianni F. Senju A. Gergely G. Csibra G. (2011). Automated gaze-contingent objects elicit orientation following in 8-month-old infants. *Dev. Psychol.* 47 1499–1503 10.1037/a0025659 21942669 PMC4636044

[B6] Farroni T. Csibra G. Simion F. Johnson M. H. (2002). Eye contact detection in humans from birth. *Proc. Natl. Acad. Sci. U.S.A.* 99 9602–9605 10.1073/pnas.152159999 12082186 PMC123187

[B7] Farroni T. Johnson M. H. Brockbank M. Simion F. (2000). Infants’ use of gaze direction to cue attention: The importance of perceived motion. *Vis. Cogn.* 7 705–718 10.1080/13506280050144399

[B8] Farroni T. Massaccesi S. Johnson M. H. (2004). Gaze following in newborns. *Infancy* 5 39–60 10.1207/s15327078in0501_2

[B9] Fiser J. Aslin R. N. (2002). Statistical learning of new visual feature combinations by infants. *Proc. Natl. Acad. Sci. U.S.A.* 99 15822–15826 10.1073/pnas.232472899 12429858 PMC137800

[B10] Gredebäck G. Fikke L. Melinder A. (2010). The development of joint visual attention: a longitudinal study of gaze following during interactions with mothers and strangers. *Dev. Sci.* 13 839–848 10.1111/j.1467-7687.2009.00945.x 20977555

[B11] Gredebäck G. Theuring C. Hauf P. (2008). The microstructure of infants’ gaze as they view adult shifts in overt attention. *Infancy* 13 533–543 10.1080/15250000802329529

[B12] Hollich G. J. Hirsh-pasek K. Golinkoff R. M. Rebecca J. Brown E. Chung H. L. (2000). Breaking the language barrier: an emergentist coalition model for the origins of word learning. *Monogr. Soc. Res. Child Dev.* 65 1–123 10.1111/1540-5834.00091 12467096

[B13] Hood B. M. Willen J. D. Driver J. (1998). Adult’s eyes trigger shifts of visual attention in human infants. *Psychol. Sci.* 9 131–134 10.1111/1467-9280.00024

[B14] Houston-Price C. Nakai S. (2004). Distinguishing novelty and familiarity effects in infant preference procedures. *Infant Child Dev.* 13 341–348 10.1002/icd.364

[B15] Houston-Price C. Plunkett K. Duffy H. (2006). The use of social and salience cues in early word learning. *J. Exp. Child Psychol.* 95 27–55 10.1016/j.jecp.2006.03.006 16677668

[B16] Hunter M. A. Ames E. W. (1988). A multifactor model of infant preferences for novel and familiar stimuli. *Adv. Infancy Res.* 5 69–95 10.1037/0012-1649.19.3.338

[B17] Jasso H. Triesch J. Deák G. Lewis J. M. (2012). A unified account of gaze following. *IEEE Trans. Auton. Ment. Dev.* 4 257–272 10.1109/tamd.2012.2208640

[B18] Johnson M. H. Dziurawiec S. Ellis H. Morton J. (1991). Newborns’ preferential tracking of face-like stimuli and its subsequent decline. *Cognition* 40 1–19 10.1016/0010-0277(91)90045-6 1786670

[B19] Johnson S. Slaughter V. Carey S. (1998). Whose gaze will infants follow? The elicitation of gaze-following in 12-month-olds. *Dev. Sci.* 1 233–238 10.1111/1467-7687.00036

[B20] Kanda T. Hirano T. Eaton D. Ishiguro H. (2004). Interactive robots as social partners and peer tutors for children: a field trial. *Hum. Comput. Interact.* 19 61–84 10.1207/s15327051hci1901&2_4

[B21] Kirkham N. Z. Slemmer J. A. Johnson S. P. (2002). Visual statistical learning in infancy: evidence for a domain general learning mechanism. *Cognition* 83 B35–B42 10.1016/S0010-0277(02)00004-5 11869728

[B22] Kirkham N. Z. Slemmer J. A. Richardson D. C. Johnson S. P. (2007). Location, location, location: development of spatiotemporal sequence learning in infancy. *Child Dev.* 78 1559–1571 10.1111/j.1467-8624.2007.01083.x 17883448

[B23] Moore C. Angelopoulos M. Bennett P. (1999). Word learning in the context of referential and salience cues. *Dev. Psychol.* 35 60–68 10.1037/0012-1649.35.1.60 9923464

[B24] Moore C. Corkum V. (1994). Social understanding at the end of the first year of life. *Dev. Rev.* 14 349–372 10.1006/drev.1994.1014

[B25] Morissette P. Ricard M. Decarie T. G. (1995). Joint visual attention and pointing in infancy: a longitudinal study of comprehension. *Br. J. Dev. Psychol.* 13 163–175 10.1111/j.2044-835X.1995.tb00671.x

[B26] O’Connell L. Poulin-Dubois D. Demke T. Guay A. (2009). Can infants use a onhuman agent’s gaze direction to establish word–object relations? *Infancy* 14 414–438 10.1080/15250000902994073 32693449

[B27] Reid V. M. Striano T. (2005). Adult gaze influences infant attention and object processing: implications for cognitive neuroscience. *Eur. J. Neurosci.* 21 1763–1766 10.1111/j.1460-9568.2005.03986.x 15845105

[B28] Reid V. M. Striano T. Kaufman J. Johnson M. H. (2004). Eye gaze cueing facilitates neural processing of objects in 4-month-old infants. *Neuroreport* 15 2553–2555 10.1097/00001756-200411150-00025 15538194

[B29] Roder B. J. Bushnell E. W. Sasseville A. M. Marie A. (2000). Infants’ preferences for familiarity and novelty during the course of visual processing. *Infancy* 1 491–507 10.1207/S15327078IN0104_9 32680296

[B30] Senju A. Csibra G. (2008). Gaze following in human infants depends on communicative signals. *Curr. Biol.* 18 668–671 10.1016/j.cub.2008.03.059 18439827

[B31] Senju A. Johnson M. H. Csibra G. (2006). The development and neural basis of referential gaze perception. *Soc. Neurosci.* 1 220–234 10.1080/17470910600989797 18633789

[B32] Striano T. Chen X. Cleveland A. Bradshaw S. (2006). Joint attention social cues influence infant learning. *Eur. J. Dev. Psychol.* 3 289–299 10.1080/17405620600879779

[B33] Triesch J. Teuscher C. Deák G. O. Carlson E. (2006). Gaze following: why (not) learn it? *Dev. Sci.* 9 125–147 10.1111/j.1467-7687.2006.00470.x 16472311

[B34] Varga Jakobsen K. Frick J. E. Simpson E. A. (2013). Look here! the development of attentional orienting to symbolic cues. *J. Cogn. Dev.* 14 229–249 10.1080/15248372.2012.666772

[B35] Wahl S. Michel C. Pauen S. Hoehl S. (2012). Head and eye movements affect object processing in 4-month-old infants more than an artificial orientation cue. *Br. J. Dev. Psychol.* 31 212–230 10.1111/bjdp.12001 23659892

[B36] Wronski C. Daum M. M. (2014). Spatial orienting following dynamic cues in infancy: grasping hands versus inanimate objects. *Dev. Psychol.* 50 2020–2029 10.1037/a003715 24932723

[B37] Wu R. Gopnik A. Richardson D. C. Kirkham N. Z. (2011). Infants learn about objects from statistics and people. *Dev. Psychol.* 47 1220–1229 10.1037/a0024023 21668098

[B38] Wu R. Kirkham N. Z. (2010). No two cues are alike: depth of learning during infancy is dependent on what orients attention. *J. Exp. Child Psychol.* 107 118–136 10.1016/j.jecp.2010.04.014 20627258

[B39] Yoshida H. (2012). A cross-linguistic study of sound symbolism in children’s verb learning. *J. Cogn. Dev.* 13 232–265 10.1080/15248372.2011.573515 23807870 PMC3691963

[B40] Yu C. Smith L. B. (2013). Joint attention without gaze following: human infants and their parents coordinate visual attention to objects through eye-hand coordination. *PLoS ONE* 8:e79659 10.1371/journal.pone.0079659 PMC3827436 24236151

